# Needle-stick injuries in Isfahan, Iran: quality improvement

**DOI:** 10.1186/1753-6561-5-S6-P221

**Published:** 2011-06-29

**Authors:** S Mobasherizadeh, A Ebneshahidi, M Rahimi, M Ostadrahimi, GR Masoumi

**Affiliations:** 1 Isfahan University of Medical Sciences, Tehran, Iran, Islamic Republic Of; 2 Research & development, sadi Hospital, Isfahan, Tehran, Iran, Islamic Republic Of; 3 Research & Development Pol Ideal Pars, Tehran, Iran, Islamic Republic Of

## Introduction / objectives

The goal of the study was to investigate needle-stick injuries among health care workers in Isfahan, Iran and to evaluate the preventive measures which are to be taken to reduce these injuries and to improve their safety quality.

## Methods

This study was carried out before and after intervention, and took six years, 2003 to2008. At the beginning of the study, the injuries resulting from sharps among Isfahan Province health care workers in 31 hospitals were investigated.Then in the second, third and fourth years of intervention plan, the first phase was carried out by using the data analysis information. Most of the intervention was instructional and some parts were by using appropriate equipment. The third phase was the evaluation of intervention measures. Data were analyzed by Excel software and SPSS_13_.

## Results

The rate of needle-stick injuries was 61.4% in 2003 from which 25.5% were injured at least twice during that year. Most cases of injuries 36.6% were among personnel recapping the needles. In the third phase, the injuries were reduced to 7%(p<0.001) and only 3% of the injured staff were injured twice and the injuries resulting from recapping were reduced to 11.3%.The injuries’ average per each staff in the first year was 1.27 which was reduced to 0.2%(p<0.001)in the sixth year of the study.

## Conclusion

The study shows significant reduction in needle stick injuries after intervention.However, providing a purposeful plan according to existing demands and problems in hospitals of each area, along with continuous training programsneeds appropriate supervision and safer medical devices utilization, which have the minimum risk of injury and which can considerably minimize the risk of injuries among health care workers.

## Disclosure of interest

None declared.

